# PvGAP: Development of a globally-applicable, highly-multiplexed microhaplotype amplicon panel for *Plasmodium vivax*

**DOI:** 10.1101/2025.04.30.25326751

**Published:** 2025-05-02

**Authors:** Alfred Hubbard, Edwin Solares, Lauren Bradley, Brook Jeang, Delenasaw Yewhalaw, Daniel Janies, Eugenia Lo, Guiyun Yan, Elizabeth Hemming-Schroeder

**Affiliations:** 1 Department of Epidemiology, Johns Hopkins Bloomberg School of Public Health, Baltimore, Maryland, USA; 2 Department of Computer Science and Engineering, UC San Diego, San Diego, California, USA; 3 Department of Population Health & Disease Prevention, UC Irvine Wen School of Population and Public Health, Irvine, California, USA; 4 School of Medical Laboratory Sciences, Jimma University, Jimma, Ethiopia; 5 Department of Bioinformatics and Genomics, University of North Carolina at Charlotte, Charlotte, North Carolina, USA; 6 Department of Microbiology & Immunology, Drexel University, Philadelphia, Pennsylvania, USA; 7 College of Veterinary Medicine and Biomedical Sciences, Colorado State University, Fort Collins, Colorado, USA

## Abstract

*Plasmodium vivax* malaria research has yet to fully benefit from the advances in genomic surveillance that have revolutionized *P. falciparum* epidemiology. Closing this gap is critical because genomic tools are necessary to achieve certain malaria control program objectives: 1) they allow monitoring of the spread of drug resistance and thus selection of therapeutic drugs based on local prevalence of resistance; 2) they permit classification of infections as local or imported, enabling more precise targeting of control resources; and 3) they can distinguish reinfection, recrudescence, and relapse, a necessity for conducting therapeutic efficacy studies. To achieve these objectives, microhaplotype marker panels that allow powerful genotyping of polyclonal infections are needed. A handful of such panels have been published for *P. vivax*, but they may be limited to certain geographic areas. We here present a Globally-applicable Amplicon Panel for *P. vivax* (PvGAP) designed to maximize discriminatory capability between geographic regions. PvGAP has 80 high diversity targets suitable for population genomics and eight targets of specific epidemiological interest, such as putative markers of drug resistance. We demonstrate PvGAP achieves robust amplification with field data and that it clearly distinguishes samples from different locations both at a regional and global scale. PvGAP is ready for broad application that can support powerful and comprehensive studies of malaria genomic epidemiology.

## Background

*Plasmodium vivax* is the most widely distributed malaria parasite, and there is increasing evidence that *P. vivax* is circulating in all regions of Africa, despite malaria control program efforts to reduce morbidity and mortality caused by *P. falciparum* (Twohig et al., 2019). In addition to causing considerable morbidity, including anemia, malnutrition, and poor school performance in early childhood, *P. vivax* can cause severe and life-threatening malaria (Anstey et al., 2012). In part because *P. vivax* has historically been considered benign or non-fatal, *P. vivax* malaria remains understudied in comparison to *P. falciparum* malaria. Improving our understanding of *P. vivax* epidemiology is a key step to planning effective antimalarial interventions and improving malaria control.

Population genomics is one powerful approach for gaining insights into malaria epidemiology and informing control programs. Genomic information from malaria parasites can be used to target control resources to areas of high transmission and evaluate the effectiveness of antimalarial interventions with genetic indicators of transmission intensity (Neafsey et al., 2021). However, the capacity of population genomics for investigating malaria epidemiology is limited by technical constraints and costs. For example, classical biallelic SNP assays have low sensitivity to detect multiple parasite strains and parasite diversity within a host (Koepfli & Mueller, 2017). On the other hand, whole genome sequencing (WGS) provides high resolution data, but the data is costly to produce (Tessema et al., 2022) and store (Neafsey et al., 2021). One cost-effective method for obtaining moderately high-resolution genomic data with high sensitivity to detecting within-host parasite diversity is targeted deep sequencing of genetically diverse and informative amplicons (Koepfli & Mueller, 2017; Tessema et al., 2022).

Furthermore, data generated from genotyping-by-sequencing can be used to assess multiallelic microhaplotypes, genetic loci small enough to be sequenced as one target that contain two or more SNPs. The major advantage of this approach is that microhaplotype markers have been shown to provide higher power for relatedness inference than biallelic SNPs, particularly in the case of infections that are polyclonal, meaning they have multiple, genetically-distinct parasite strains (Tessema et al., 2022). For researchers that study eukaryotic parasite epidemiology, highly-multiplexed amplicon sequencing panels of polymorphic microhaplotype markers along with advances in analytical methods present a promising avenue to accurately assessing *Plasmodium* genetic relatedness and transmission patterns (LaVerriere et al., 2022; Tessema et al., 2022).

Thus far, three amplicon sequencing panels have been designed and validated with field data for *P. vivax* (Kattenberg et al., 2022; Kleinecke et al., 2024; Popkin-Hall et al., 2024), and the authors are aware of at least two more efforts currently in development. Both the Kattenberg et al. (2022) and Popkin-Hall et al. (2024) panels mostly target SNPs, but they do contain 11 (Kattenberg et al., 2022) and 25 (Popkin-Hall et al., 2024) gene targets focused on putative markers of drug resistance or, in the case of Popkin-Hall et al. (2024), vaccine candidates. While genotyping such genes is also of great interest for malaria control purposes, these genes may be under selection and thus are not suitable for population genetics analyses that aim to reveal information about patterns of transmission. The Popkin-Hall et al. (2024) panels are impressive in size (1200 SNPs between three panels), which may counterbalance the power constraints of SNPs in many settings, but the technology used is still fundamentally limited in the presence of polyclonal infections. The Kleinecke et al. (2024) panel is promising for population genetics with highly polyclonal data: while it has one marker for species identification and four putative markers of drug resistance, the other 93 targets are high diversity sites intended for genetic relatedness analyses. However, this panel was designed with data predominately from Southeast Asia and Oceania (Siegel et al., 2024) and thus may be most appropriate for studying *P. vivax* epidemiology in those regions.

This paper describes the design and validation of a new *P. vivax* Globally-applicable Amplicon Panel (PvGAP) of microhaplotype targets. The panel possesses similar properties to those of the Kleinecke et al. (2024) panel: there are 80 high diversity markers for population genetics, and eight gene targets that include a section of *pvdbp* (*P. vivax* Duffy binding protein) and seven putative drug resistance markers. However, PvGAP and its associated protocol presented here differ from the Kleinecke et al. (2024) panel in three key aspects: 1) PvGAP was designed using a relatively even distribution of genomes originating from South America, Southeast Asia, and Africa, as opposed to primarily genomes from Southeast Asia and Oceania (Siegel et al., 2024); 2) genetic distance between genomes was considered in marker selection for PvGAP, as opposed to considering genetic diversity alone; and 3) the protocol for PvGAP uses a non-proprietary library preparation workflow with primers designed to minimize cross-reactivity as opposed to relying on the proprietary rhAmpSeq platform to minimize mispriming (Integrated DNA Technologies, Newark NJ). Combined, the first two distinctions may make PvGAP better suited to differentiate infections between regions. The ability to identify imported infections and determine their geographic origin is one of the main control applications for malaria genomics (Neafsey et al., 2021). The third distinction allows the PvGAP protocol to be less expensive and allows more flexible modification of the reagents without risking primer interaction. Altogether, these unique aspects of PvGAP make this a powerful and flexible new tool for *P. vivax* population genomics that should be suitable for a variety of epidemiological applications, including geographic assignment of imported infections.

## Methods

### Panel design

Whole genome sequence data for 198 *P. vivax* isolates from eight countries (Cambodia, the China-Myanmar border region, Colombia, Ethiopia, Madagascar, Malaysia, Panama, and Peru) were downloaded from NCBI (Table 1). Sequences were aligned by bwa (Li & Durbin, 2009) in conjunction with SAMtools (Danecek et al., 2021). Alignments were removed using BCFTools (Danecek et al., 2021) if they showed multiple mappings, mappings across chromosomes, had mean coverage > 4x, or quality values < 20. SNPs and indels were called using GATK (Van der Auwera & O’Connor, 2020) in conjunction with Picard-tools (*Picard Toolkit*, 2019) and VCF-tools (Danecek et al., 2011). Final SNPs and indels were called using the HaplotypeCaller and GenotypeGVCT algorithms.

To identify candidate markers for our amplicon sequencing panels, we used a sliding window method adapted from the approach used by Tessema et al. (2022) to design a microhaplotype panel for *P. falciparum*. We divided the genome into 200bp sliding windows every 100bp, yielding a total of 242,135 windows, using the sliding.window.transform function in the PopGenome R package (Pfeifer et al., 2014). This initial set of windows was then filtered based on presence of tandem repeats, presence of indels, and genetic diversity. Tandem repeats in the genome were identified using Tandem Repeats Finder (Benson, 1999). Windows that contained tandem repeats > 40 bp, dinucleotide repeats > 8 bp, homopolymer repeats > 8 bp, or trinucleotide repeats > 12 bp were removed. Second, windows containing any insertion or deletion from variant calling were removed. Third, within-country nucleotide diversity (π) was calculated for the remaining windows in PopGenome, and windows that were monomorphic (π = 0) in isolates from > 25% of the countries were excluded prior to further analysis, as these windows would not be informative for fine-scale genomic analyses in certain study regions. This filtering process led to 2,498 candidate windows remaining for potential inclusion in the panel.

We proceeded to evaluate and compare candidate windows based on polymorphism and genetic structuring. Specifically, we evaluated windows for mean within-country nucleotide diversity (π) and averaged fixation index (F_ST_) for each country against all other individuals. Both values were calculated in PopGenome using the F_ST.stats function. Windows were then ranked by their F_ST_ and π values. To achieve a relatively even distribution of loci across the genome, for each chromosome the 10 windows with the highest π values, the window with the highest mean F_ST_ value, and the window with the highest π that also was in the highest 8% of F_ST_ values were selected. After that, the remaining windows were ranked overall (i.e., all chromosomes pooled together), and the 72 windows with the highest π value, the 28 windows with the highest F_ST_ value, and the 10 windows with the highest π that were also in the highest 8% of F_ST_ values were selected. At this point, windows were removed from consideration if there was insufficient availability of conserved regions outside of the target window for primer design and replaced with the window having the next highest value.

The final set of candidate windows which were selected for primer design consisted of 278 targets with the number of targets per chromosome ranging from 16 to 25. The minimum value for targets selected for π was 0.0024 and for F_ST_ was 0.50. These minimum values were among the top 55% and 93% of values, respectively, of the original 2,498 candidate windows.

Our goal was to generate a panel with approximately 100 targets, but we selected an abundance of potential targets expecting fall-out from primer incompatibilities during primer design and uneven amplification and/or sequencing coverage during assay development. The 278 targets were submitted to GTseek LLC (Twin Falls, ID; https://gtseek.com) for primer design with the goal of designing primers that generate minimal crosstalk during multiplexed PCR reactions (Campbell et al., 2015). Primers were successfully designed for 179 of the 278 targets. After small test sequencing runs, we further removed primers for targets that did not amplify consistently, yielding a reduced set of 80 targets.

In addition to the targets selected for their π and/or F_ST_ values, eight additional loci of interest were added to the panel, bringing the final panel size to 88. These additional loci target *pvdbp* and putative markers of drug resistance (Table 2). Primers for these targets were also designed by GTSeek LLC to minimize crosstalk among primers during amplification.

### Panel evaluation with field samples

Two groups of analyses were performed with field samples to evaluate panel performance. A smaller group of samples, six dried blood spot (DBS) samples and four whole blood samples, were processed with a variety of laboratory methods to determine the best wet lab workflow, with respect to on-target reads, target amplification, and costs. This analysis, along with the results and selected protocol, is described in detail in Supplemental Text 1. A larger group of DBS samples was used to evaluate the panel’s ability to measure population genetic metrics of epidemiological importance. The subsections below describe the analysis of this larger set of field samples.

### Sample collection

The field samples were gathered as part of an ongoing sub-Saharan Africa International Center for Excellence for Malaria Research (ICEMR) project (Githure et al., 2022; Yan et al., 2022). Capillary blood samples were obtained through both passive case detection (i.e., clinical malaria cases) and community cross-sectional surveys (i.e., subclinical malaria infections). Samples were obtained from all consenting individuals residing in two locations in the Oromia regional state of Southwestern Ethiopia: the Arjo-Didessa sugarcane plantation and Gambella rice development areas described in previous studies (Getachew et al., 2023; Githure et al., 2022; Yan et al., 2022). Capillary blood samples (300 μL) were preserved on filter paper as dried blood spots (DBS) and stored with desiccant. At the time of collection, individuals were also screened for *Plasmodium* infection by Pf/Pv (HRP2/pLDH) Ag Combo RDT test kits (Access Bio Ethiopia, INC.). Study participants who were malaria positive by RDT tests during the survey were directed to seek appropriate treatment at the nearby health facility. Ethical approval was obtained from the institutional review boards at the University of California at Irvine; Case Western Reserve University, Cleveland, OH; and the Institute of Health of Jimma University, Ethiopia. Permission was obtained from the local health authorities to conduct the demographic surveillance and parasitological mass blood surveys. All residents willing to participate in the study were included; adults provided signed consent for themselves and assent for minors under 18 years of age after explanation of the study objectives and methodologies. Confidentiality of the study participants’ information was maintained.

### Library preparation and sequencing

DNA was extracted from DBS samples using the Saponin/Chelex method (Bereczky et al., 2005). *Plasmodium* species were detected and quantified by qPCR assays with standard curves as previously described (Bradley et al., 2023). Duffy genotypes were determined for individuals with *Plasmodium* infections by sequencing a region of the human DARC gene as previously described (Bradley et al., 2023). *P. vivax* infections from Duffy negative individuals were excluded from the analyses in this manuscript as these samples were differentially processed in an attempt to increase sequencing coverage from extremely low parasite density infections; these results and analyses related to Duffy genotype will be presented in a separate manuscript. The library preparation protocol is described in detail in Supplemental Text 2. In brief, it consists of selective whole genome amplification (SWGA) to increase the density of parasite DNA in the sample, adapted from Oyola et al. (2016) and Cowell et al. (2017); followed by an adapted version of the GT-seq PCR protocol to affix primers (see Campbell et al. (2015) for the original technique and LaVerriere et al. (2022) for a previous application to *P. falciparum*); followed by application of Nate’s Plates kits (GTseek LLC) to affix dual indexing tags and normalize sequence quantity across samples. Various QC assays are performed after each step and on the final libraries, as described in Supplemental Text 2. Sequencing was performed on an Illumina MiSeq, using the Reagent Kit v3 and 10% PhiX spike ins. Samples were amplified and sequenced in duplicate.

### Bioinformatics analysis

The malaria amplicon pipeline published alongside LaVerriere et al. (2022) was used to extract and filter microhaplotypes, using primer and reference files appropriate for our *P. vivax* panel. This pipeline uses the Divisive Amplicon Denoising Algorithm (DADA2; Callahan et al., 2016) as the core method for identifying and filtering amplicons, and then applies a handful of postprocessing steps to remove likely sequencing artifacts and chimeras.

Replicate read counts were analyzed in two different ways. To assess panel performance, mean read counts for each sample and locus were computed across replicates (Supplemental Figure 1). To compare sequencing yield with parasitemia, the total read count was calculated for each replicate, and the mean of these totals was computed for each sample.

### Panel evaluation with MalariaGEN data

#### Data preparation

To assess the utility of the panel at a coarser spatial scale, whole genome sequences from the MalariaGEN Pv4 project (MalariaGEN et al., 2022) for the years 2015 through 2016 were downloaded for comparison with our results. These are the two most recent years in the dataset that have more than 50 samples.

Variants and samples that failed the QC process of the MalariaGEN authors were removed. Samples with an *F*_*WS*_ below 0.95 were also removed, as these are considered to be polyclonal (Auburn et al., 2012). Finally, samples from longitudinal studies and returning travelers were removed. This yielded a final analysis set of 185 samples, from 10 different countries and 19 distinct sites.

The remaining variants were filtered to the genomic regions represented in the panel using bcftools view (Danecek et al., 2021). Haplotype sequences were obtained for each sample and locus using bcftools consensus (Danecek et al., 2021), using the PvP01 reference genome (Auburn et al., 2016).

#### Population genetics

Haplotype sequences for each locus were aligned using the MUSCLE algorithm with the R package msa (Bodenhofer et al., 2015), after which selection was assessed with Tajima’s D (Tajima, 1989), calculated with the R package pegas (Paradis, 2010).

Selection was assessed separately for each population in the dataset, using the population definitions provided by the MalariaGEN authors. Pairwise linkage disequilibrium (LD) between all pairs of loci that share a chromosome was estimated with the <mi> statistic (Agapow & Burt, 2001), calculated with the poppr R package (Kamvar et al., 2014). The *p*-value thresholds for both the tests of selection and pairwise LD were corrected for multiple testing using the Bonferroni method. Loci under significant selection or in significant LD with other loci, at the 0.05 level, were filtered out before proceeding with the analyses described below.

Nei’s expected heterozygosity (Nei, 1978) was estimated for each locus with more than one allele using the poppr R package (Kamvar et al., 2014). This method is simpler than that used with the field data above, which is made possible by the absence of polyclonal “infections” in this virtual dataset. This metric was computed separately for each country and for each site in Cambodia and Vietnam, to facilitate comparison with the relatedness analysis (see below). In each case, *t*-tests were performed between each pair of groups to identify significant differences. The Bonferroni method was used to correct for multiple testing. At the country level, countries with fewer than 15 samples were removed to avoid introducing bias from low sample size.

Identity-by-descent (IBD) between samples was estimated using the R package Dcifer (Gerlovina et al., 2022). This tool uses population-level allele frequencies for each individual to estimate whether observed sharing of genotypes between sample pairs is because of sharing in the most recent common ancestor (in which case they are said to be identical-by-descent) or due to chance alone. Significance is assessed using a likelihood ratio approach.

Relatedness values estimated with Dcifer were analyzed in two ways: at the country level for the entire dataset, and at the site level for Cambodia and Vietnam. In both cases, the mean relatedness of all constituent sample pairs was computed for each pair of countries or sites. In addition, the fraction of highly-related pairs was computed for the site level comparison, using a relatedness threshold of 0.25, as metrics based on the number of highly-related pairs are more sensitive to recent gene flow (Taylor et al., 2017). As with the expected heterozygosity analysis, countries with fewer than 15 samples were removed.

## Results

### Panel characteristics

**Figure 1:**
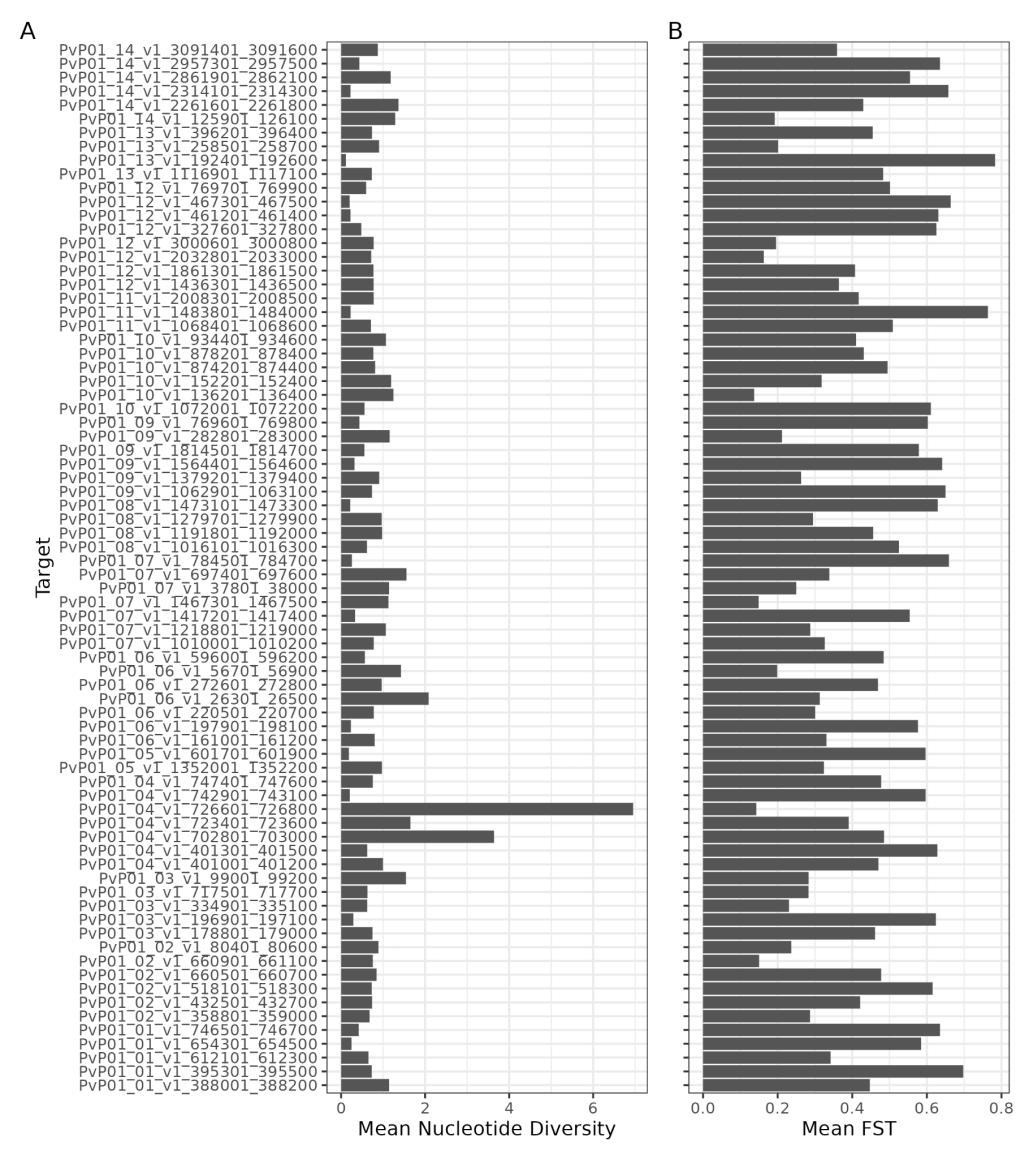
Mean nucleotide diversity **(A)** and mean F_ST_ **(B)** for the genome windows selected for inclusion in the final panel.

As intended, the genome windows selected for inclusion in the final panel all have high nucleotide diversity (Figure 1A) and/or high F_ST_ (Figure 1B), making them informative for *P. vivax* genomic epidemiology analyses.

### Panel evaluation with field samples

As stated in Methods, the smaller set of field samples used to optimize the laboratory protocol is described in Supplemental Text 1. The results below pertain to the larger set of field samples.

Mean read counts across replicates (Supplemental Figure 1) indicate that overall amplification was quite good, with most samples yielding 10–1000 reads for most loci. A handful of samples consistently did not amplify well, and the *pvdbp* locus also consistently did not amplify well. This locus did perform well in the smaller dataset used for protocol optimization, however.

**Figure 2:**
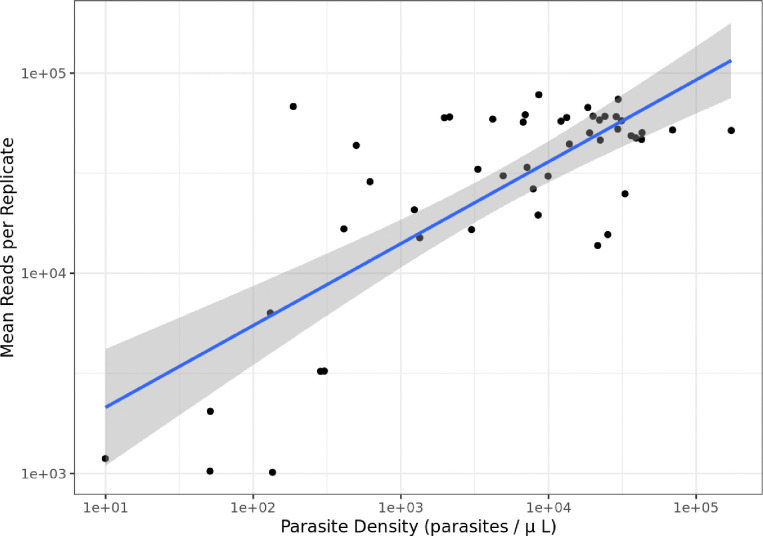
Scatterplot showing the relationship between parasite density and mean total read count (i.e., the mean reads obtained for each sample) for the field isolates. Both axes are log_10_ scaled. The blue line is a simple linear regression, with shaded areas showing 95% confidence intervals.

Mean total read counts, computed across replicates, remain fairly high at low parasite densities (Figure 2), suggesting the SWGA protocol is delivering good amplification even for low parasitemia samples. Amplification does become unreliable around 100 parasites/μL.

### Panel evaluation with MalariaGEN data

In the analysis of Pv4 samples, no pairs of loci from the same chromosome were identified as being in significant LD, after the significance threshold of 0.05 was Bonferroni-corrected. However, two loci had a negative Tajima’s *D* in one or more populations at the 0.05 significance level, after Bonferroni correction. These loci, Pvcrt_o.10k.indel and PvP01_10_v1_1072001_1072200, were removed from the dataset prior to genetic diversity and relatedness analysis.

In terms of genetic diversity, Colombia and Indonesia had a lower overall expected heterozygosity than the other countries (Supplemental Figure 2A), and Ho Chi Minh had the lowest expected heterozygosity of any of the sites in Cambodia and Vietnam (Supplemental Figure 2B). However, none of these differences were significant after applying the Bonferroni correction for multiple testing.

**Figure 3:**
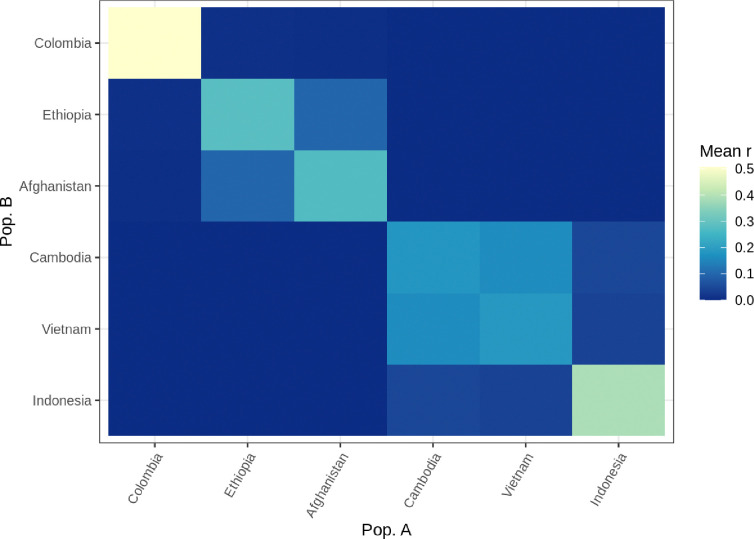
Heatmap showing the mean relatedness of all constituent sample pairs in each pair of countries in the MalariaGEN data. Color swatches along the diagonal indicate within-country relatedness. Countries with fewer than 15 samples have been removed.

Most sample pairs have a relatedness of zero (Supplemental Figure 3A). Among the other pairs, there are a few clonal samples (relatedness of one) and most of the rest have a relatedness below 0.25 (the relatedness of half-siblings; Supplemental Figure 3B). As expected, mean IBD-based relatedness tends to be higher within countries than between countries (Figure 3). Also, relatedness between countries clearly shows regional divisions, particularly with Southwest Asia/East Africa and Southeast Asia (Figure 3). Gene flow appears to be particularly high between Cambodia and Vietnam, as the relatedness between these two countries is comparable to the relatedness within each country.

**Figure 4:**
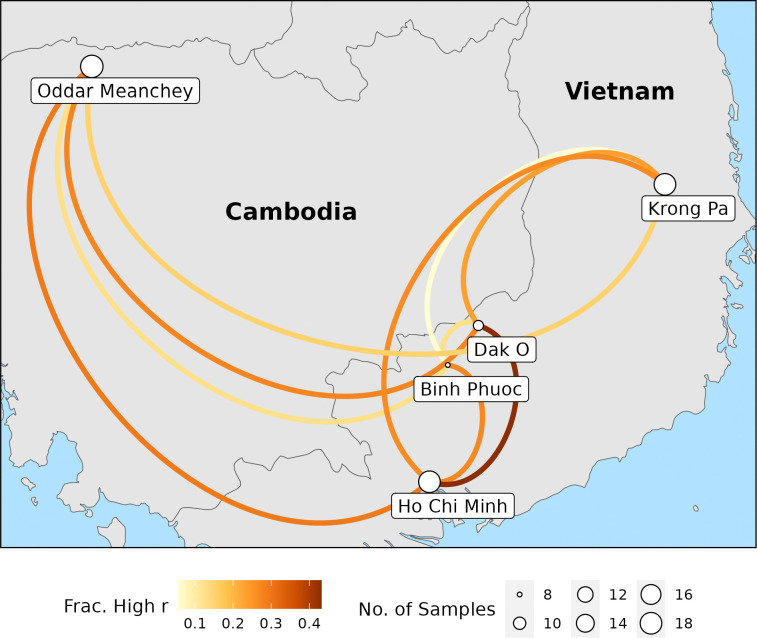
Network showing between-site relatedness (visualized as the color of the links) for samples from Cambodia and Vietnam. Between-site relatedness was calculated as the fraction of highly-related sample pairs. Node size is scaled according to the number of samples from that site.

To demonstrate the panel’s ability to distinguish patterns of genetic connectivity within a region, the results for Cambodia and Vietnam are examined in detail. At the site level, mean relatedness of constituent sample pairs provides some ability to discriminate gene flow patterns between pairs of sites (Supplemental Figure 4A), but the distinctions between sites become clearer when the fraction of highly-related sample pairs is used instead (Supplemental Figure 4B). This is consistent with the theoretical expectation that metrics based on highly-related sample pairs will perform better as analysis moves from the global to the local level, as these metrics are better equipped to capture recent gene flow (Taylor et al., 2017). However, even when the fraction of highly-related sample pairs is considered, the patterns of genetic relatedness in this region are complex. When visualized in geographic space, it becomes apparent that a simple pattern of isolation-by-distance does not explain genetic relatedness of *P. vivax* in this region (Figure 4). Instead, it can be seen that Ho Chi Minh and Dak O have comparatively high relatedness to the other sites, implying that these locations may be hubs of malaria transmission in the region.

## Discussion and Conclusion

The microhaplotype marker panel designed and evaluated in this study shows substantial promise for enhancing understanding of genomic epidemiology in *P. vivax* in a variety of geographic regions. After several filters, we obtained a final panel with 88 loci total, 80 of which are designed for population genetics and 8 of which are genes of epidemiological interest (e.g., potential drug resistance/tolerance markers). Our evaluation with field samples shows that the panel works well with DBS samples from symptomatic and asymptomatic individuals, yielding consistently high read counts except in the presence of rather low parasitemia. The population genetics analysis with MalariaGEN data demonstrates that the panel not only distinguishes between geographic regions, but also can identify substantial within-region variation in genetic relatedness, as evidenced by the analysis of sites in Cambodia and Vietnam.

Microhaplotype panels such as the one described in this paper over several advantages for genomic epidemiology studies. They have greater sensitivity for detecting minority clones than WGS, while costing substantially less per sample (Tessema et al., 2022). Also, they offer enhanced power to distinguish related and unrelated pairs of infections if the samples are polyclonal (Tessema et al., 2022). Finally, the enhanced sensitivity for minority clones combined with multiallelic data allows more accurate estimation of complexity of infection (COI) in high transmission settings (Tessema et al., 2022).

These characteristics support a variety of public health applications. First, accurate measurements of COI enable experiments that correlate within-host parasite diversity with the entomological inoculation rate, the gold standard for measuring transmission. Such experiments are necessary to establish whether genetic metrics such as COI serve as a better proxy for transmission than case incidence. Second, sensitive and consistent detection of minority clones is necessary to distinguish recrudescence, reinfection, and relapse (Auburn et al., 2021), which in turn is necessary to identify treatment failure in therapeutic efficacy studies. Finally, more powerful estimation of genetic relatedness between malaria infections enables discrimination of local and imported cases. Distinguishing the source of cases permits National Malaria Control Programs to prioritize limited resources to either suppress local transmission or test and treat returning travelers (Wesolowski et al., 2018).

Though there are other *P. vivax* microhaplotype panels, the panel described in this paper presents certain advantages over other offerings. While it is relatively similar to the Kleinecke et al. (2025) panel, it may be more appropriate for a wider variety of applications, as steps were taken in our panel design process to identify markers with high power to differentiate the geographic origin of infections across three continents. The MIP panels presented in Popkin-Hall et al. (2024) may perform as well as microhaplotypes in certain settings, but the MIP technology has limited sensitivity to genotype low parasitemia infections (Neafsey et al., 2021). Once all microhaplotype panels for *P. vivax* currently under development are published, it will be important to undertake a rigorous assessment of all panels using simulated datasets to understand which combination of markers from the various panels performs best in different situations.

Library preparation costs for our panel are estimated to be roughly 13 USD per sample, not including primers, which are typically a one-time purchase. Kleinecke et al. (2025) estimates roughly 14–28 USD (converted from AUD) per sample, depending on whether the PCR reaction volume is halved to save money. Thus, costs are theoretically comparable between the panels. However, as our primers were designed to minimize crosstalk and we are not reliant on the proprietary rhAmpSeq platform, the reagents used in our protocol (e.g., the master mix) are more flexible. Also, the targeted pre-amplification procedure described in Supplemental Text 1 could be used in place of SWGA for high parasitemia samples to considerably reduce the costs of library preparation.

A potential limitation of this work is that the relatedness patterns observed in the WGS-based microhaplotypes may be confounded by the fact that the data was aggregated from multiple contributing studies, each with its own sample collection strategy. This could be particularly true in Southeast Asia, where the Ho Chi Minh and Oddar Meanchey samples came from one study (1128-PV-MULTI-GSK) and Binh Phuoc, Dak O, and Krong Pa came from another (1157-PV-MULTI-PRICE; MalariaGEN et al., 2022). Using data simulated in a consistent manner, as part of the future work on comparing panels described above, would resolve this limitation.

The panel described in this paper constitutes an important step forward for *P. vivax*: a large panel of microhaplotype loci selected for high diversity *and* differentiation, ideal for genomic epidemiology at a global scale. Given the growing need for cost-effective yet powerful tools to measure the complexities of *P. vivax* malaria epidemiology, this panel has the potential to dramatically enhance the surveillance of *vivax* malaria and thus accelerate progress towards elimination.

## Supplementary Material

Supplement 1

Supplement 2

Supplement 3

## Figures and Tables

**Table 1:** *P. vivax* genomes used in panel design

Country	No. of Genomes	Reference

Cambodia	31	Parobek et al. (2016)
China-Myanmar	28	Chen et al. (2017)
Colombia	8	Winter et al. (2015)
Ethiopia	33	Auburn et al. (2019) Lo et al. (2019)
Madagascar	9	Menard et al. (2013)
Malaysia	30	Auburn et al. (2018)
Panama	29	Accession: PRJNA655141
Peru	30	Cowell et al. (2018)

**Table 2:** *P. vivax* genes of particular epidemiological interest

Gene name	Gene ID	Chromosome	Start*	End*

*pvdbp*	PVP01_0623800	PvP01_06_v1	983467	983623
*pvcrt*	PVP01_0109300	PvP01_01_v1	442085	442281
*pvdhfr*	PVP01_0526600	PvP01_05_v1	1077441	1077602
*pvdhps*	PVP01_1429500	PvP01_14_v1	1270726	1270916
*pvdhps*	PVP01_1429500	PvP01_14_v1	1270343	1270530
*pvk13*	PVP01_1211100	PvP01_12_v1	485037	485238
*pvk13*	PVP01_1211100	PvP01_12_v1	486524	486684
*pvmdr1*	PVP01_1010900	PvP01_10_v1	479798	479986

* Start and end coordinates are zero-based
