# Supplementary material for: PvGAP: Development of a globally-applicable, highly-multiplexed microhaplotype amplicon panel for Plasmodium vivax
Source: medRxiv. 2025 May 2:2025.04.30.25326751. Preprint. [Version 1] doi: 10.1101/2025.04.30.25326751 (PMC12060969; doi:10.1101/2025.04.30.25326751)
Supplement: Supplement 2 [file media-2.docx]

**Step 1: Parasite DNA enrichment and preparation**

sWGA protocols adapted from:

- <https://doi.org/10.1186/s12936-016-1641-7>
- <https://doi.org/10.1128/mBio.02257-16>

**Step 1.1: Selective whole-genome amplification (sWGA)**

**PCR recipe:**

| Component | Vol. (uL) |
| --- | --- |
| Phi29 reaction buffer | 5 |
| dNTPs | 5 |
| Albumin | 2.5 (of 1:10 dilution) |
| Phi29 polymerase | 3 |
| Primers | 1.25 ul (of 100 uM primer) |
| gDNA | 10 |
| NF dH20 | *Adjust* |
| Total Volume | **50** |

**PCR cycling condition:**

| Temp. (°C) | Time |
| --- | --- |
| 35 | 5:00 |
| 34 | 10:00 |
| 33 | 15:00 |
| 32 | 20:00 |
| 31 | 30:00 |
| 30 | 16:00:00 |
| 65 | 15:00 |
| 4 | Hold |

**Step 1.2: Bead cleanup of sWGA products:**

***NOTE: when discarding supernatant in the following steps, visually inspect pipette tip to ensure you are not discarding magnetic beads!***

1. Transfer 20ul of sWGA product to a new plate
2. Add 36ul of [AmpureXP, CleanNGS, or Takara Nucleomag] beads (for a 1:1.8 PCR product to beads ratio); mix well and incubate for 15 min at RT.
3. Briefly centrifuge plate; move plate to magnetic rack and let sit for 5 min at RT.
4. Remove supernatant and discard while being careful not to discard any beads
5. While on magnetic rack, add 100 ul of 80% EtOH (freshly prepared); incubate for 30 s and discard.
6. Repeat wash step.
7. Remove plate from magnetic rack and incubate uncovered for 10 min at RT to dry beads. If there is residual liquid after 10 minutes, carefully remove with a pipette tip and discard.
8. Resuspend beads into 20ul (starting volume) of TE buffer; mix well and incubate for 5 min at RT
9. Briefly centrifuge plate; move plate to magnetic rack and let sit for 3 min at RT
10. Collect 20 ul of supernatant in a new plate

*Optional: confirm successful sWGA by PCR/qPCR of parasite targets and/or by DNA quantification by fluorometer/spectrophotometer. Compare pre-sWGA DNA to cleaned up sWGA product on all products or a representative sample*

**Step 2: GT-seq PCR**

GT-seq protocol adapted from:

- <https://doi.org/10.1111/1755-0998.12357>
- <https://doi.org/10.1111/1755-0998.13622>

**Step 2.1: PCR 1**

Thaw components at RT. Mix well and centrifuge briefly.

**PCR recipe:**

| Component | Vol. (uL) |
| --- | --- |
| Qiagen Plus MM (2X) | 3.5 |
| 0.25 uM primer pool | 1.5 |
| sWGA product | 2.0 |
| Total Volume | 7.0 |

*To make primer pool: Add 2uL of each primer (suspended at 200 uM) into total volume of 1600 uL with TE buffer to get a 0.25 uM final concentration in the pool of nested primers.*

**PCR cycling condition:**

| Step | Temp. (°C) | Time | Cycles |
| --- | --- | --- | --- |
| Hot Start | **95** | 15:00 | 1 |
| Denaturation | **95** | 0:30 |  |
| Annealing | **57** | 0:30 | 5 |
| Extension | **72** | 2:00 |  |
| Denaturation | **95** | 0:30 |  |
| Annealing | **65** | 0:30 | 15 |
| Extension | **72** | 0:30 |  |
| Hold | **10** | Hold | 1 |

Slow cool: 5% ramp rate (~ 0.1-0.3 deg/s)

**Step 2.2: Dilute**

Dilute PCR product 1:4 by adding 14ul Nuclease-free H_2_0 to each well; mix well

**Step 2.3: QC**

Confirm successful PCR by running a pool of representative sample of diluted PCR products on a 1.5% agarose gel. For example, for each plate, take 1ul of samples from 3 rows, pool them, and run the pool on a lane of the gel. Save the gel with unused lanes for later.

**Step 2.4: Remove remaining primers and primer dimers by bead cleanup**

Pull beads from refrigerator 30 minutes prior to use to allow them time to warm to RT.

***NOTE: when discarding supernatant in the following steps, visually inspect pipette tip to ensure you are not discarding magnetic beads!***

1. You should have ~20 uL of diluted PCR product. Add 14 uL of SPRI beads for a 1:0.7 ratio; mix well and incubate at RT for 5 minutes.
2. Move tube to magnetic rack and let sit for 3 minutes.
3. Remove and discard supernatant
4. While on magnetic rack, add 200 uL of 75% EtOH (freshly prepared) to tube, incubate for 30 seconds and discard.
5. Repeat wash step.
6. Remove tube from magnetic rack and incubate for 10 min with open lid at RT. If there is residual liquid after 10 minutes, carefully remove with a pipette tip and discard.
7. Resuspend beads into 20 uL of TE buffer
8. Incubate for 5 min at RT
9. Move tubes to magnetic rack and let sit for 3 min at RT
10. Collect cleared supernatant in a new tube

**Step 3: Nate’s Plates by GTseek**

A PCR-based normalization kit designed to incorporate dual indexing tags and return equal numbers of sequencing library constructs from each sample. This is a patent-pending protocol draft.

**Step 3.1: PCR 2**

Thaw Qiagen MM at RT. Mix well and centrifuge briefly.

1. Add 2ul Qiagen Plus MM and 2ul of Nuclease-free H_2_0 to each well of Nate’s Plate
2. Transfer 1uL diluted product from Step 2.5 to Nate’s Plate.
3. Place Nate’s Plate in thermocycler

**PCR cycling condition:**

| Step | Temp. (°C) | Time | Cycles |
| --- | --- | --- | --- |
| Hot Start | **95** | 15:00 | 1 |
| Denaturation | **94** | 0:30 |  |
| Annealing | **57** | 0:30 | 2 |
| Extension | **72** | 2:00 |  |
| Denaturation | **94** | 0:30 | 18 |
| Extension | **72** | 0:45 |  |
| Extension | **72** | 2:00 | 1 |
| Hold | **4** | Hold | 1 |

**Step 3.2: Sample normalization - Bind**

1. Pool samples from each well in Nate’s Plate and collect in tube; label tube as POOL. If doing multiple plates, pool each plate separately for downstream QC.
2. Add 500 uL of Nate’s Plates Bead Buffer (2X) to POOL tube. Vortex the tube of Streptavidin beads and ensure they are adequately homogenized.
3. Carefully transfer 1 uL of strep beads to POOL tube. Vortex and incubate POOL for 12 minutes at RT, inverting tube occasionally.
4. Place POOL tube on magnetic rack and incubate for 3 minutes.
5. Remove and discard supernatant, being careful not to disturb the bead pellet.

***NOTE: the bead pellet will be very small and difficult to see. Like a wisp of dirt. Ensure that you can see the bead pellet at every step.***

1. While on magnetic rack, add 1 ml of Nate’s Plates Wash buffer; incubate for 1 minute; and discard supernatant.
2. Repeat wash step.
3. Resuspend beads in 20 uL sterile nuclease-free water; mix well

**Step 3.3: Sample normalization – Release**

1. Pipette 10uL of resuspended beads into a strip tube for thermal cycling and add the following components

| Component | Vol. (uL) |
| --- | --- |
| Qiagen Plus MM (2X) | 20.0 |
| 10X Bead Release primers | 4.0 |
| Nuclease-free water | 6.0 |
| Resuspended beads | 10.0 |
| Total Volume | 40.0 |

*Optional: The remaining 10 uL of resuspended beads can be stored at -20 if desired.*

1. Place strip tube in thermocycler

**PCR cycling condition:**

| Step | Temp. (°C) | Time | Cycles |
| --- | --- | --- | --- |
| Hot Start | **95** | 15:00 | 1 |
| Denaturation | **94** | 0:30 |  |
| Annealing | **60** | 0:30 | 6 |
| Extension | **72** | 0:30 |  |
| Extension | **72** | 2:00 | 1 |
| Hold | **4** | Hold | 1 |

**Step 3.4: Bead size selection**

***NOTE: when discarding supernatant in the following steps, visually inspect pipette tip to ensure you are not discarding magnetic beads!***

1. Transfer PCR product to a new tube and place on magnetic rack. Let sit for 3 minutes
2. Transfer 25 uL cleared supernatant to new tube. Add 20 uL SPRI beads; mix well and incubate at RT for 5 minutes.

***NOTE: Optionally save remaining ~15 uL of cleared supernatant for storage and/or electrophoresis***

1. Move tube to magnetic rack and let sit for 3 minutes.
2. Remove and discard supernatant
3. While on magnetic rack, add 200 uL of 75% EtOH (freshly prepared) to tube, incubate for 30 seconds and discard.
4. Repeat wash step.
5. Remove tube from magnetic rack and incubate for 10 min with open lid at RT. If there is residual liquid after 10 minutes, carefully remove with a pipette tip and discard.
6. Resuspend beads into 15ul of TE buffer
7. Incubate for 5 min at RT
8. Move tubes to magnetic rack and let sit for 3 min at RT
9. Collect cleared supernatant in a new tube
10. Add 1.5 uL elution buffer (EB) with 1% Tween-20

**Step 4: QC the libraries**

**Step 4.1: Determine concentration of library using Qubit HS DNA assay and record concentration in ng/µl.**

1. Make sure you click “Calculate Stock” on the Qubit, specify the number of µl you added, and change the units to ng/µl.
2. Record concentration in ng/ul
3. Dilute library to ~2ng/µl in a minimum of 30µl.

**Step 4.2: Visually confirm amplicon sizes are as expected and adapter dimers have been removed by bead size selection**

1. Run diluted libraries on an agarose gel
2. If significant adapter dimers remain, repeat bead size selection step

***NOTE: adapter dimers will occur at around 150 bp. Target amplicon sizes should fall in the 220-530 bp range (amplicon size range + adapter lengths)***

**Step 4.3: qPCR**

1. In new tubes, prepare two 1:100 dilution of your diluted ~1 ng/ul library using 1X TE buffer with 0.1% Tween-20.
2. Perform a serial dilution for each first dilution, making a second 1:100 dilution for a final dilution of 1:10,000
3. Load the two 1:10,000 dilutions in your qPCR plate using your favorite flavor of qPCR for quantitation of NGS libraries (e.g. Kapa Quant Kit or NEBNext Library Quant Kit)

**Step 4.4: Pool plates at equal concentrations and submit to sequencing facility for QC (by tapestation or bioanalyzer) and sequencing**

1. Concentration of final pooled library should be 5 nM or higher
2. Submit at least 15 ul for sequencing
