# Supplementary material for: PvGAP: Development of a globally-applicable, highly-multiplexed microhaplotype amplicon panel for Plasmodium vivax
Source: medRxiv. 2025 May 2:2025.04.30.25326751. Preprint. [Version 1] doi: 10.1101/2025.04.30.25326751 (PMC12060969; doi:10.1101/2025.04.30.25326751)
Supplement: Supplement 3 [file NIHPP2025.04.30.25326751v1-supplement-3.pdf]

medRxiv preprint doi: https://doi.org/10.1101/2025.04.30.25326751; this version posted May 2, 2025. The copyright holder for this preprint (which was not certified by peer review) is the author/funder, who has granted medRxiv a license to display the preprint in perpetuity.
It is made available under a CC-BY 4.0 International license .

Supplemental Material

Figures

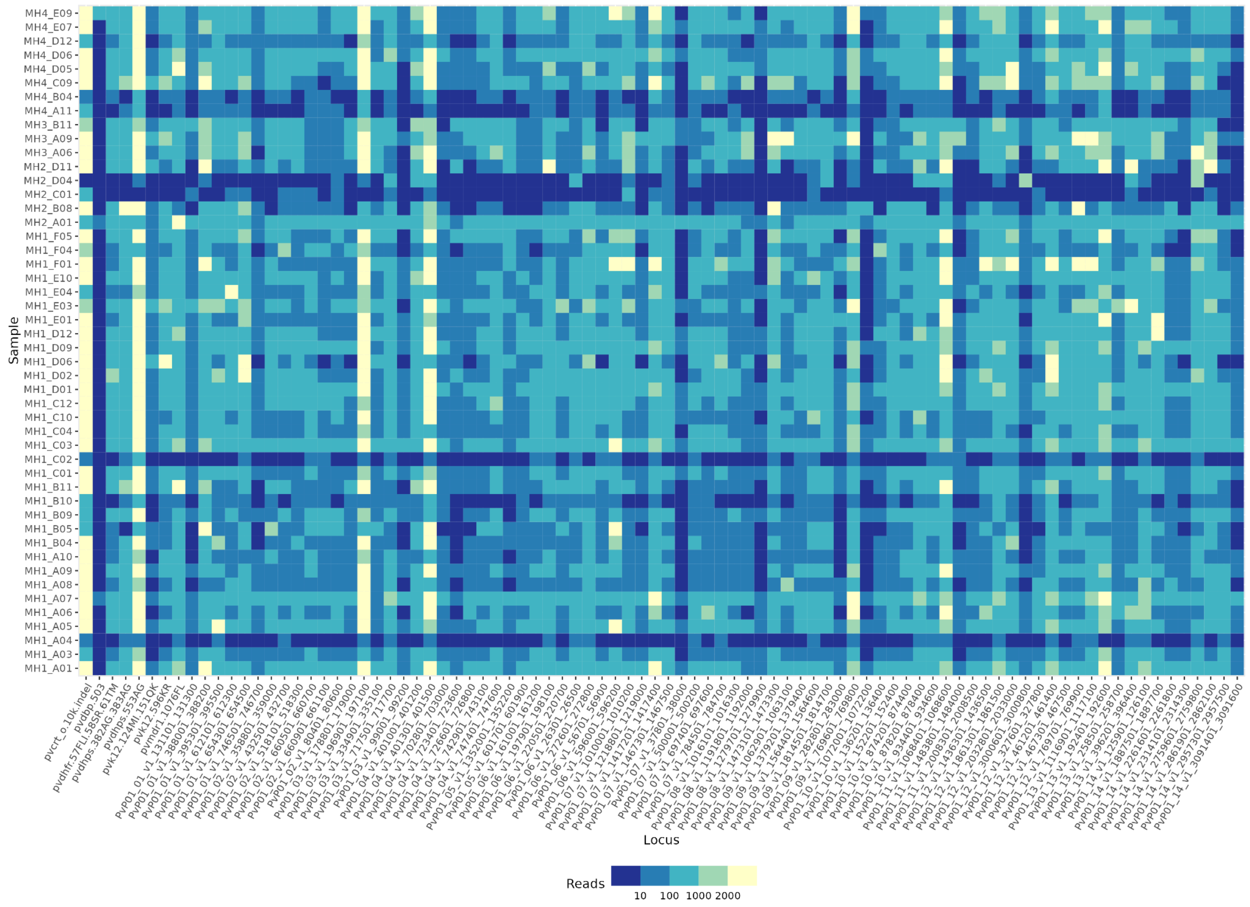

Supplemental Figure 1: Mean read counts across replicates for the field isolates, visualized separately for each locus.

medRxiv preprint doi: https://doi.org/10.1101/2025.04.30.25326751; this version posted May 2, 2025. The copyright holder for this preprint
(which was not certified by peer review) is the author/funder, who has granted medRxiv a license to display the preprint in perpetuity.
It is made available under a CC-BY 4.0 International license .

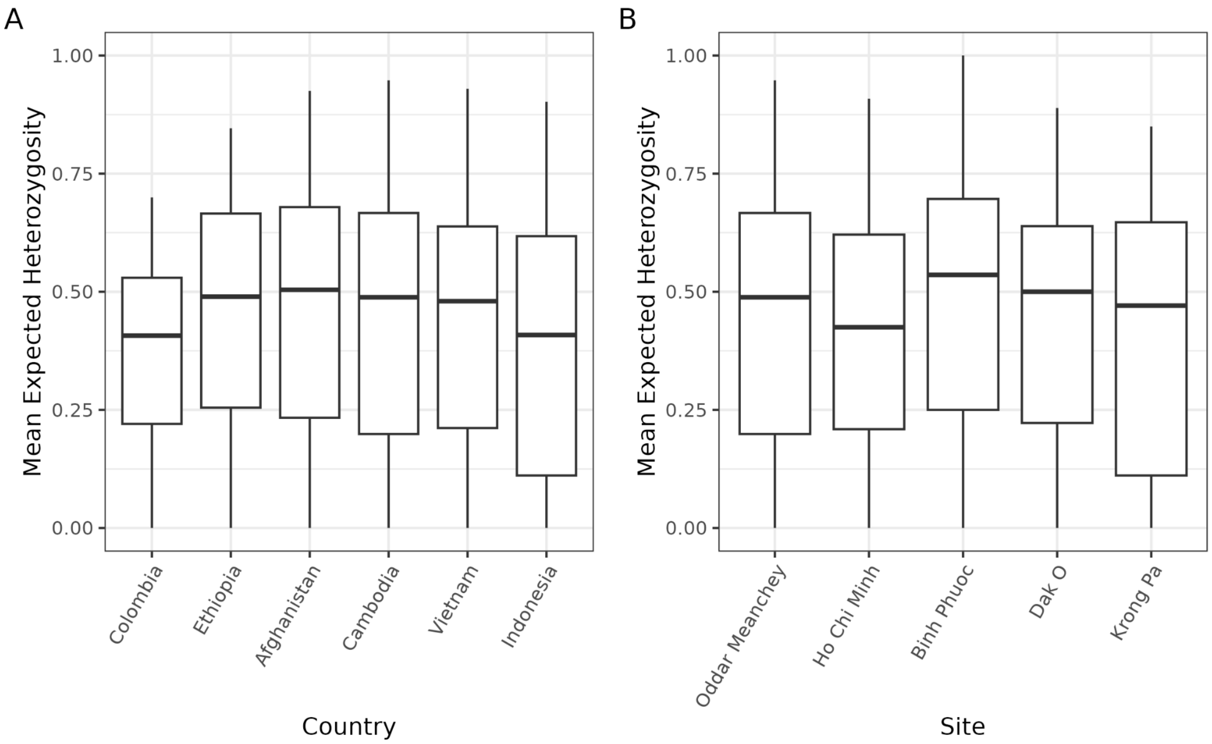

Supplemental Figure 2: Distribution of mean expected heterozygosities for each marker, separated by country **(A)** and site **(B)**. The whiskers are Tukey-style and extend to a maximum of 1.5 * IQR.

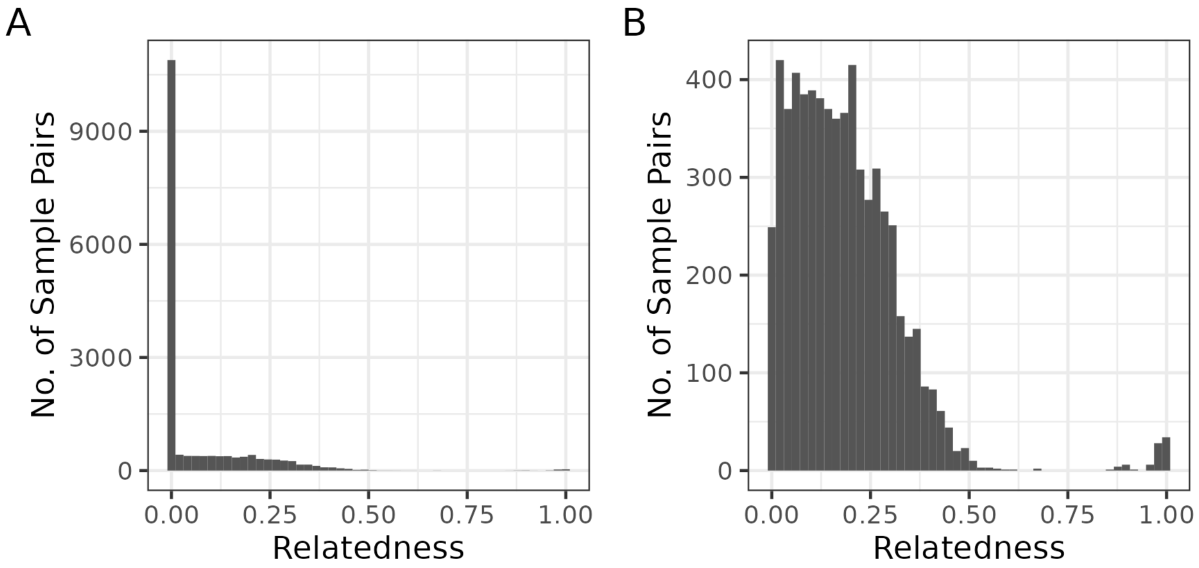

medRxiv preprint doi: https://doi.org/10.1101/2025.04.30.25326751; this version posted May 2, 2025. The copyright holder for this preprint (which was not certified by peer review) is the author/funder, who has granted medRxiv a license to display the preprint in perpetuity.
It is made available under a CC-BY 4.0 International license .

Supplemental Figure 3: Histograms displaying relatedness estimated by Dcifer for **(A)** all sample pairs and **(B)** sample pairs with relatedness greater than zero.

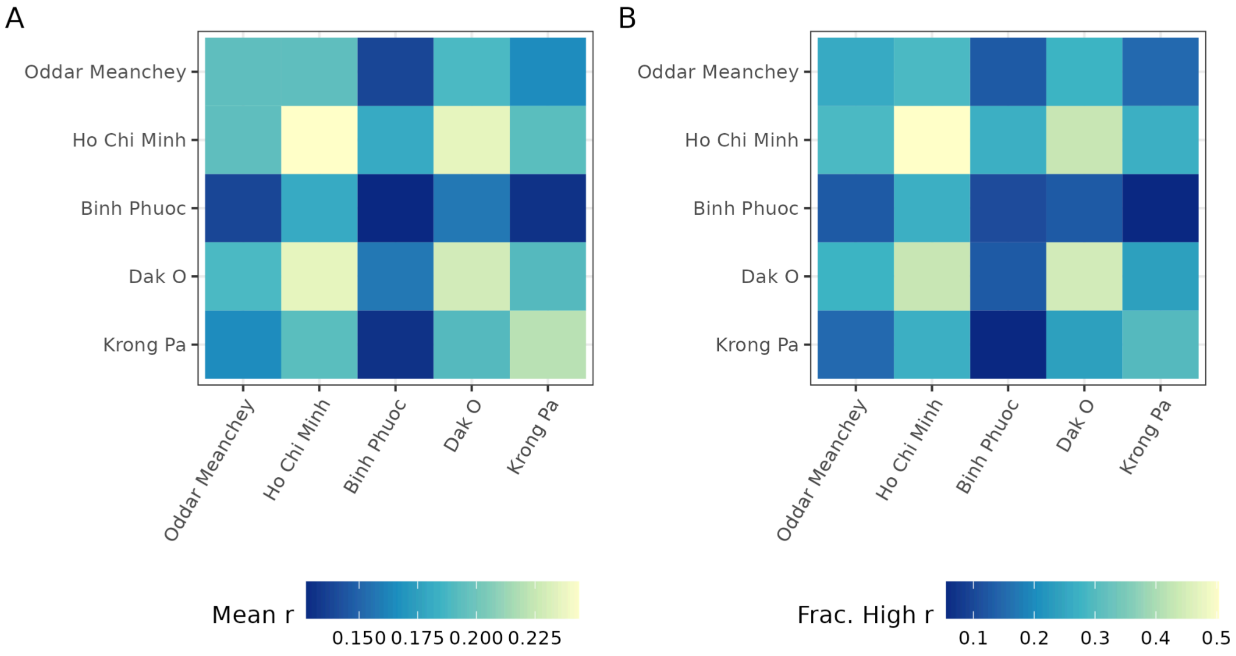

Supplemental Figure 4: Heatmaps showing the relatedness within and between sites in Cambodia and Vietnam. **(A)** shows the mean relatedness of all constituent sample pairs and **(B)** gives the fraction of highly-related sample pairs corresponding to each pair of sites. Color swatches along the diagonal indicate within-site relatedness.
